# Advances in Non-Electrochemical Sensing of Human Sweat Biomarkers: From Sweat Sampling to Signal Reading

**DOI:** 10.3390/bios14010017

**Published:** 2023-12-28

**Authors:** Mingpeng Yang, Nan Sun, Xiaochen Lai, Xingqiang Zhao, Wangping Zhou

**Affiliations:** 1 School of Automation, Nanjing University of Information Science and Technology, 219 Ningliu Road, Nanjing 210044, Chinazxq@nuist.edu.cn (X.Z.); 2 Jiangsu Collaborative Innovation Centre on Atmospheric Environment and Equipment Technology, Nanjing University of Information Science and Technology, 219 Ningliu Road, Nanjing 210044, China

**Keywords:** human sweat, non-electrochemical sensing, wearable sensors, microfluidic sampling

## Abstract

Sweat, commonly referred to as the ultrafiltrate of blood plasma, is an essential physiological fluid in the human body. It contains a wide range of metabolites, electrolytes, and other biologically significant markers that are closely linked to human health. Compared to other bodily fluids, such as blood, sweat offers distinct advantages in terms of ease of collection and non-invasive detection. In recent years, considerable attention has been focused on wearable sweat sensors due to their potential for continuous monitoring of biomarkers. Electrochemical methods have been extensively used for in situ sweat biomarker analysis, as thoroughly reviewed by various researchers. This comprehensive review aims to provide an overview of recent advances in non-electrochemical methods for analyzing sweat, including colorimetric methods, fluorescence techniques, surface-enhanced Raman spectroscopy, and more. The review covers multiple aspects of non-electrochemical sweat analysis, encompassing sweat sampling methodologies, detection techniques, signal processing, and diverse applications. Furthermore, it highlights the current bottlenecks and challenges faced by non-electrochemical sensors, such as limitations and interference issues. Finally, the review concludes by offering insights into the prospects for non-electrochemical sensing technologies. By providing a valuable reference and inspiring researchers engaged in the field of sweat sensor development, this paper aspires to foster the creation of innovative and practical advancements in this domain.

## 1. Introduction

**Figure 1:**
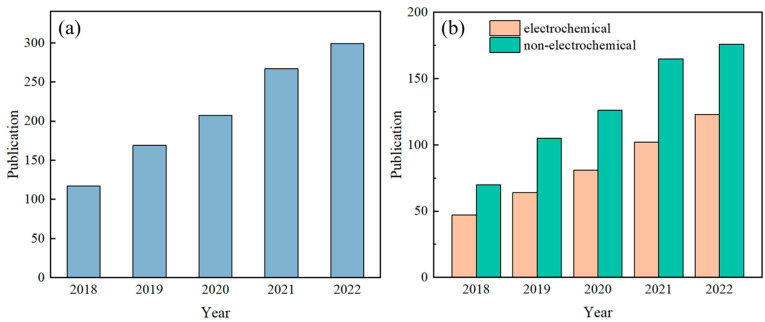
Number of published papers on sweat sensors in the past five years. Subfigure (**a**) presents the overall trend of the total number of papers, while subfigure (**b**) specifically compares the number of papers on electrochemical sweat sensors and non-electrochemical sweat sensors. Note: The quantity of articles pertaining to sweat sensors in Figure (**a**) was determined by conducting a search on the Web of Science database using the keywords “wearable sensor” and “sweat”. In Figure (**b**), the number of published articles on electrochemical methods for measuring sweat was obtained through a search on the Web of Science using the keywords “wearable sensor”, “sweat”, and “electrochemical”. The count of articles on non-electrochemical methods for sweat detection was derived by subtracting the number of electrochemical sweat sensor articles from the total number of sweat sensor articles.

Sweat contains a variety of biomarkers, including metabolites [1], electrolytes [2], trace elements [3], and small amounts of macromolecules [4], and there is a strong correlation between human sweat and blood [5,6,7]. Analyzing sweat offers a non-invasive means for health assessments and disease diagnoses. For instance, cystic fibrosis can be diagnosed based on sweat chloride concentration [8], and indirect assessment of blood glucose concentration can be made based on sweat glucose concentration [9], while the hydration status of the human body can be determined based on the concentration of potassium and sodium ions in sweat [10]. As such, detecting sweat biomarkers has garnered significant attention in recent years, yielding impressive research outcomes. The number of published papers related to wearable sensors and sweat, retrieved using the keywords “wearable sensor” and “sweat” from the Web of Science database, has shown a steady increase over the past five years (Figure 1).

Researchers have conducted substantial work on sweat detection, resulting in various detection methods. Electrochemical sensors convert the chemical components in sweat through an “ion–electron” conversion interface, converting ion or macromolecule information in sweat (including type and concentration) into specific electrical signals (usually current and voltage) [11,12,13]. Therefore, they serve as a bridge between sweat and electrical signals and represent the mainstream detection method for sweat sensors [14,15,16]. These sensors can catalyze macromolecules in sweat into their metabolites, generating electronic signals that can characterize the concentration of the target analytes. This method is called voltammetry. They can also transform ion concentration information into potential signals via the ion–electron conversion interface, known as potentiometry. In the context of sweat sensors, the measurement of electrical signals from the electrodes typically involves the utilization of a flexible printed circuit board (FPCB) integrated with electronic components [17]. To fulfill the requirements for signal processing in wearable sweat sensors and communication with smart terminal devices, this flexible circuit must encompass essential functionalities, including signal acquisition, conditioning, transmission, and power management [18,19,20]. Nevertheless, the relatively large size of the FPCB can lead to wearer discomfort when connected to microfluidic components and worn on the body surface [21,22]. Moreover, the incorporation of measurement circuits introduces cost implications, making sensor reuse (or component reuse) a notable concern. Overcoming challenges such as electrical signal crosstalk and battery life is also crucial. Significantly, it should be noted that potential-type sensors are prone to signal drift and necessitate additional calibration prior to usage [23,24].

Despite the considerable attention paid by scholars to electrochemical methods for detecting sweat components, not all sweat sensors use electrochemical methods. Other detection methods may have advantages that electrochemical methods lack as described above and are also worthy of our attention. These methods are referred to as non-electrochemical methods and, in many cases, they can complement electrochemical methods and are more suitable for detecting biomarkers in sweat [25,26,27]. Sweat sensors based on non-electrochemical methods primarily rely on changes in optical wavelength or intensity to measure the concentration of target substances in sweat [28,29,30].

Fresh and abundant sweat is a prerequisite for accurate measurement by sensors. In the early days, sweat was collected by tying gauze to the skin, and the collected sweat was centrifuged and analyzed using desktop equipment [31,32,33]. This method was laborious and time consuming. With the development of microfluidic technology, various methods are now used to collect sweat. For example, paper-based materials have many capillaries and hydrophilic groups, allowing sweat to spontaneously transfer and flow in a specified direction when the paper is cut into a specific shape [34,35,36,37,38]. Additionally, the wax printing process can be used to construct microchannels on the paper surface by printing hydrophobic wax onto filter paper. After baking at high temperatures, the hydrophobic substance infiltrates the tiny gaps in the paper, blocking the flow of the liquid, and sweat flows along the unprinted area. Thread, which is composed of multiple thin threads wound together and has many capillaries, has stronger toughness and can withstand greater stress compared to paper-based materials. Moreover, thread is a key material in embroidery technology. When stitched into fabric using embroidery techniques, it can fix the components of the device and transmit fluids [39,40,41,42,43]. Microfluidic valves can precisely control sweat and guide it into multiple microchambers for timed sampling analysis, achieving high-precision detection of sweat target molecules [44,45,46].

In electrochemical sweat sensors, microfluidic components are required to be in close contact with the electrodes, and the sensing electrodes need to be connected to the circuit module. On the other hand, non-electrochemical sweat sensors do not necessitate the integration of microfluidic components with electronic devices. Instead, they utilize imaging devices, like smartphones, to capture images of the detection area and analyze information, such as color and absorption wavelength, to quantitatively determine the concentration of the target substance [47,48]. Non-electrochemical detection methods can be classified into several categories, including colorimetric methods, fluorescence methods, surface-enhanced Raman spectroscopy (SERS) methods, and visual semi-quantitative reading methods. Colorimetry [49,50,51] entails the mixing of a color reagent with the sample, where the reagent reacts with specific target molecules. The resulting change in color of the reagent is subsequently analyzed to determine the concentration of the target substance. However, it should be noted that colorimetry has inherent limitations in terms of detection accuracy and is particularly vulnerable to external environmental interference. Fluorescence methods [52,53] involves mixing a specific fluorescent dye with the target substance, and using an external light source to excite the mixture, causing the fluorescence intensity of the mixture to change. This change can be analyzed to quantify the concentration of the target substance. Fluorescence has high sensitivity and can achieve high-precision measurements. SERS methods [54,55] involve the analysis of the surface-enhanced Raman spectra of the analyte to determine its concentration. This technique exhibits high sensitivity and stability, enabling the detection of very low concentrations of the target substance. Furthermore, the human eye can serve as a natural camera. Visual semi-quantitative readings [56,57] by the human eye can be obtained without the need for external electronic devices. For instance, the scale on the surface of the microfluidic device can be read directly with the naked eye, and the color of the detection area can be compared with a standard reference color for semi-quantitative analysis. Electrochemical and non-electrochemical methods each have their own strengths and weaknesses, displaying a complementary trend. For clarity, we have outlined the advantages and disadvantages of these methods in detecting sweat biomarkers in Table 1.

To quantitatively analyze the captured images of the detection area, various methods have been employed to determine the concentration of the target analyte [58,59,60]. Colorimetric images can be analyzed by examining the color information, such as Red, Green, and Blue (RGB) values, to establish a linear relationship between a single RGB value and the concentration of the target analyte [61]. However, in certain cases, multiple RGB values may exhibit correlation with the target analyte, necessitating a comprehensive analysis utilizing a calibration equation established using a chromaticity diagram [62]. Additionally, external environmental factors can interfere with the detection of the target analyte, which can be mitigated by employing a calibration equation to reduce measurement errors.

Similarly, spectral information, including fluorescence intensity and reflection wavelength, is utilized in fluorescence and SERS methods for the quantitative analysis of the target analyte, offering higher sensitivity and stability [19,63,64].

This article presents a discussion and introduction of the recent advancements in non-electrochemical sweat sensors, covering sampling, detection, and signal reading methods. Sampling strategies include using paper-based, thread-based, or microfluidic valves to transport and manipulate liquids. Detection methods mainly include colorimetry, fluorescence, and other methods. For signal reading, color information (RGB values) or signal intensity in the spectrum are used for quantitative analysis. Finally, this review addresses the bottlenecks and challenges of non-electrochemical sweat sensors and provides an outlook on the future development trends for this type of sensor.

## 2. Sampling of Non-Electrochemical Sweat Sensors

### 2.1. Paper-Based Sampling of Non-Electrochemical Sweat Sensors

Paper-based materials are rich in hydrophilic groups, such as carboxyl and hydroxyl groups, and are endowed with numerous capillaries, enabling them to spontaneously transport liquids and achieve fluid flow in a specific direction without requiring external pumping [19,65,66]. Additionally, paper-based materials are cost-effective and biodegradable, thus serving as a commonly employed material for microfluidic devices.

**Figure 2:**
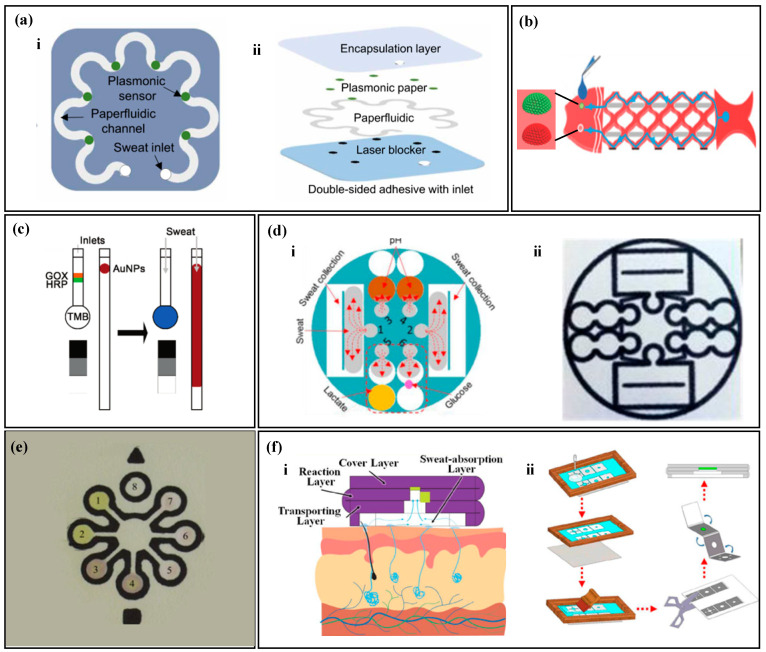
Sampling of paper-based non-electrochemical sweat sensors. (**a**) Sweat sampling using an S-shaped paper-based channel: (i) top view of the paper-based sweat sensor with an S-shaped channel; (ii) exploded view of the paper-based sweat sensor with an S-shaped channel. Reproduced with permission [68]. Copyright 2022. Reproduced with permission from the American Association for the Advancement of Science. (**b**) Sweat sampling using a fish-shaped paper-based sweat sensor. Reproduced with permission [69]. Copyright 2019 Reproduced with permission from the Wiley-Blackwell. (**c**) Sweat sampling using a paper-based sweat sensor with enzyme and colorimetric reagents separation. Reproduced with permission [70]. Copyright 2021. Reproduced with permission from the Royal Society of Chemistry. (**d**) Microfluidic strategy of paper-based sweat sensor with wax-printed carbon powder as microchannels: (i) schematic of microfluidic sweat sampling; (ii) real image. Reproduced with permission [76]. Copyright 2019. Reproduced with permission from the Royal Society of Chemistry. (**e**) Microfluidic method of paper-based sweat sensor with wax-printed ink as microchannels. Reproduced with permission [77]. Copyright 2014. Reproduced with permission from the American Chemical Society. (**f**) Microfluidic method of sweat sampling using three-dimensional folded paper: (i) cross-sectional schematic; (ii) wax-printing process flow chart. Reproduced with permission [78]. Copyright 2022. Reproduced with permission from the American Chemical Society.

Due to their soft and pliable nature, paper-based materials are easy to cut and fold [67]. Utilizing paper-cutting techniques to create geometric patterns, fluids can flow along these patterns. Geometric-patterned paper-based channels, such as S-shaped and mesh patterns, can also conform to skin deformation [68,69]. For instance, Mogera et al. [68] created an S-shaped fluid channel by cutting filter paper (Figure 2a). Liquid is conveyed to each detection area via the capillaries present in the paper. The S-shaped design enables the paper material to possess extensibility, which adapts to the curvature of the skin surface without breaking. Similarly, Gao et al. [69] cut filter paper into a fish-shaped pattern (Figure 2b), resulting in patterned filter paper with high stretchability that forms multiple branching channels. Sweat is collected at the fish tail and transferred through the fish-scale mesh structure on the fish belly, eventually converging at the monitoring area, i.e., the fisheye.

Paper-based materials contain numerous tiny capillaries, which allow for spontaneous liquid transport. The liquid transport capabilities of paper-based materials with different pore sizes vary, with smaller pore sizes resulting in weaker fluid transport and slower liquid flow rates. Vaquer et al. [70] cut filter paper into a rectangular paper strip with a circular detection area at the end (Figure 2c). Depending on the spontaneous transport of sweat, enzymes are transported to the detection area with the color reagent. Filter paper with larger pore sizes has a higher liquid flow rate, ensuring that the enzyme level reaches the detection area adequately, resulting in a higher sensitivity of the colorimetric device. The separation of enzymes from the color reagent avoids spontaneous color response between enzymes and chromogens, thereby reducing the background signal of glucose detection.

The wax printing technique is a useful method for constructing microchannels on paper-based materials by creating hydrophobic barriers [71,72]. The surface of the paper-based material can be modified by using hydrophobic substances such as wax, polydimethylsiloxane (PDMS), or graphite, which penetrate into the micro-pores of the paper during high-temperature baking to block fluid channels and form hydrophobic barriers [73,74,75]. As a result, sweat will flow along the unprinted hydrophilic areas. Zhang et al. [76] printed carbon powder on filter paper to create a microfluidic pattern (Figure 2d). Upon heating, the carbon powder penetrates the micro-pores of the paper-based material, forming hydrophobic barriers on the hydrophilic material. Colorimetric reagents are then applied to the designated area of the filter paper. When sweat glands secrete sweat, the capillary force generated by the cellulose in the filter paper absorbs and transports the sweat along the microfluidic channel to the detection area, where a colorimetric method measures the glucose, pH, and lactate of sweat. Similarly, Lopez-Ruiz et al. [77] coated ink on the surface of the filter paper to construct a microfluidic channel (Figure 2e). Weng et al. [78] used the screen-printing process to print PDMS on the surface of the filter paper and prepared a 3D microfluidic paper folding device by simple cutting and folding (Figure 2f). The device has four layers: the bottom layer collects sweat, the transmission layer delivers sweat, the reaction layer provides a reaction zone for the target substance, and the covering layer evaporates sweat and blocks contaminants. The device is used for fluorescence analysis of the target substance by taking pictures of the reaction layer with a smartphone.

Paper-based materials are characterized by numerous capillaries with different pore sizes, resulting in varying liquid flow rates. The paper can be easily modified by cutting or using the wax printing technique to create hydrophobic channels, which guide sweat to flow along the desired path. Due to their lightweight, thin, and soft properties, paper-based materials can be in close contact with the human body and resist damage caused by bending. In addition, the low cost and simple processing of paper-based materials make them widely used in the production of wearable devices.

### 2.2. Thread-Based Sampling of Non-Electrochemical Sweat Sensors

The thread is formed by wrapping multiple strands of fine wire, and the gaps between the wrapped threads can serve as capillary channels for fluid transport [79]. Compared with paper-based materials, threads have stronger toughness and can be easily obtained from natural or artificial materials. Threads have advantages, such as being lightweight, breathable, durable, and low cost, and are widely used in the field of microfluidic sensors.

**Figure 3:**
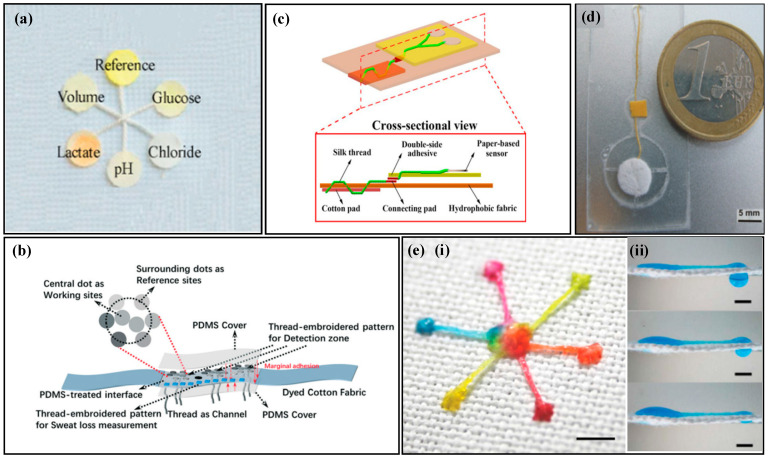
Sampling of thread-based non-electrochemical sweat sensors. (**a**) A thread-based non-electrochemical sweat sensor with hydrophilic treatment of the thread. Reproduced with permission [80]. Copyright 2020. Reproduced with permission from Elsevier. (**b**) A thread-based non-electrochemical sweat sensor that uses embroidery to fix hydrophilic threads to hydrophobic fabrics. Reproduced with permission [81]. Copyright 2021. Reproduced with permission from the Royal Society of Chemistry. (**c**) A thread-based non-electrochemical sweat sensor that uses a specially designed hydrophilic thread fixation device and fluid channel. Reproduced with permission [82]. Copyright 2020. Reproduced with permission from the Springer Nature. (**d**) A thread-based non-electrochemical sweat sensor that uses a water-absorbent pad for driving force. [83]. Copyright 2012. Reproduced with permission from Elsevier. (**e**) A super-hydrophobic textile-based sensor that is driven by surface tension to move liquid droplets (i) and the diagram of liquid flow driven by surface tension (ii). Reproduced with permission [84]. Copyright 2013. Reproduced with permission from the Royal Society of Chemistry.

The surface and fiber walls of natural cotton fibers contain wax, which cannot be wetted by liquids. Alkaline solutions can be used to remove the natural wax on the surface of the thread and enhance its hydrophilicity. Ardalan et al. [80] used a pure alkaline solution to remove the wax from the surface of the thread (Figure 3a), which served as the channel for collecting and transferring sweat in microfluidic devices. The thread was connected to the human body with medical double-sided tape, with the front end of the thread in contact with the human skin. When sweat glands secrete sweat, the thread quickly absorbs the sweat and transports it to the detection area at the end. By using embroidery techniques to connect hydrophilic threads with hydrophobic fabrics, the hydrophilic threads transport liquid, while the hydrophobic fabrics prevent the liquid in the threads from flowing outside. Zhao et al. [81] used embroidery techniques to sew hydrophilic threads into hydrophobic fabrics (Figure 3b), and fixed the colorimetric paper to the hydrophobic fabric. The hydrophilic thread collected and transported sweat to the detection area, and the hydrophobic fabric prevented the liquid in the hydrophilic thread from leaking. Since alkaline solutions may damage the fibers in the thread, Xiao et al. [82] used specially made hydrophilic threads as fluid channels (Figure 3c). Two hydrophilic threads were twisted into a Y shape, with one end connected to the sweat storage cotton pad and the other end connected to the colorimetric filter paper. The remaining end of the twisted thread was passed through the tape, connecting the sweat storage pad with other components, such as the colorimetric filter paper. Sweat was collected by the sweat storage cotton pad and transported to the colorimetric area through the twisted hydrophilic thread.

By utilizing capillary channels between tightly-wound filaments, liquids can be transported, but the pumping ability of capillary force alone is limited. Researchers use external pumps to achieve continuous flow of liquids on fabric or use surface tension at microfluidic interfaces to drive droplet movement. Curto et al. [83] use a water-absorbing pad to provide external power to thread-based microfluidic devices (Figure 3d), accelerating the flow rate of liquid inside the thread-based microfluidic device. Hydrophilic threads collect sweat and sequentially transport it to the detection pad and the water-absorbing pad. The water-absorbing pad has high water absorption and, when the liquid in the thread contacts the water-absorbing pad, it quickly absorbs the liquid from the thread and evaporates it to the outside, forming a continuous flow of liquid, which continuously updates the liquid in the detection area. Surface tension at microfluidic interfaces can drive droplets. Xing et al. [84] use the surface tension of droplets to drive them (Figure 3e). Hydrophilic threads are sewn onto hydrophobic fabric to create the inlet, fluid channel, and outlet. The inlet is double-stitched onto the fabric, while the fluid channel and outlet are single-stitched onto the fabric. The liquid is collected at the inlet and transported to the outlet. Due to the difference in surface tension between the hydrophilic and hydrophobic interface, the droplet with higher internal pressure will move towards the lower internal pressure side, i.e., the droplet moves from the inlet to the outlet, accelerating the flow of the liquid. Thread contains numerous capillaries, allowing for the transport of liquids. By sewing hydrophilic threads onto hydrophobic fabric, the thread can simultaneously fix various components of the fabric while transferring the liquid. While the capillary force of thread has limited ability to transfer liquid, it has the advantages of low cost and easy stretch, making it widely used in the field of microfluidics.

### 2.3. Sampling of Non-Electrochemical Sweat Sensors Based on Microfluidic Valves

Paper-based and thread-based sensors have advantages, such as low cost and flexibility, but they are prone to the mixture of old and new sweat, which greatly reduces the accuracy of the sensor [85,86]. With the development of microfluidics, using microvalves with different functions in microfluidic devices can control the direction of liquid flow, accurately control the sampling volume, avoid liquid mixing, and achieve timed sampling [87,88,89]. Typical microfluidic valves include capillary bursting valves (CBVs), superabsorbent polymer (SAP), and hydrophobic valves.

**Figure 4:**
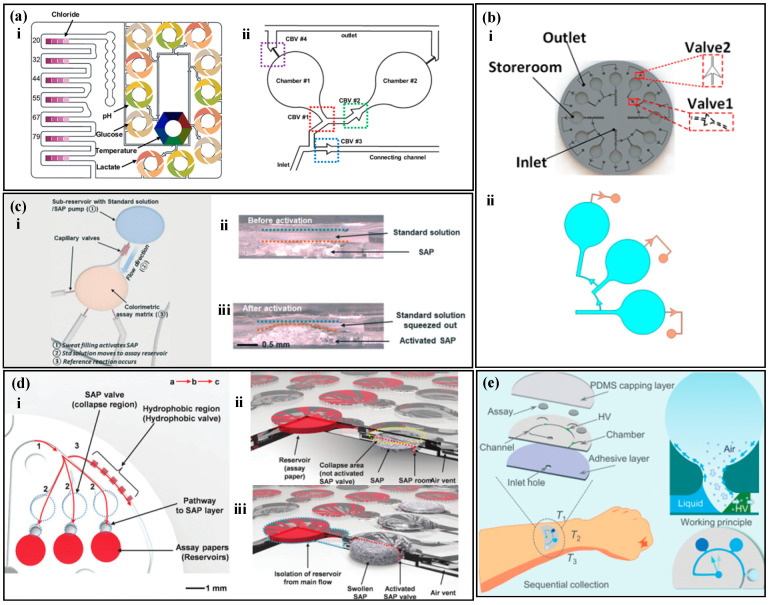
Sampling of non-electrochemical sweat sensors based on microfluidic valves. (**a**) (i) A colorimetric sweat sensor with multiple reaction zones based on multiple CBVs. (ii) A detailed view of the reaction zones, including the sampling inlet, sampling outlet, and four capillary bursting valves (CBVs). Reproduced with permission [90]. Copyright 2019. Reproduced with permission from the American Chemical Society. (**b**) (i) A colorimetric sweat sensor with multiple reaction zones based on two types of CBVs. (ii) The liquid filling three chambers was simulated in COMSOL. Reproduced with permission [91]. Copyright 2022. Reproduced with permission from the American Institute of Physics. (**c**) (i) A colorimetric sweat sensor based on SAP. (ii) Cross-sectional view before activation of the SAP valve. (iii) Cross-sectional view after activation of the SAP valve. Reproduced with permission [92]. Copyright 2020. Reproduced with permission from the Royal Society of Chemistry. (**d**) (i) A colorimetric sweat sensor based on both SAP and hydrophobic valves. (ii) Schematic diagram before activation of the SAP valve. (iii) Schematic diagram after activation of the SAP valve. Reproduced with permission [93]. Copyright 2018. Reproduced with permission from the Wiley–VCH Verlag. (**e**) A colorimetric sweat sensor based on hydrophobic valves for timed sampling. Reproduced with permission [97]. Copyright 2020. Reproduced with permission from the Royal Society of Chemistry.

CBVs are based on the surface tension of water molecules. When the microchannel of liquid flow suddenly expands, the existence of capillary resistance traps the half-moon of the liquid on the valve, and as the pressure continues to increase, when the pressure is greater than the capillary resistance, the liquid continues to flow forward, and at this time, the valve ruptures. Different valve heights, widths, and outlet angles have different burst pressures (BP), as shown in the following formula [90]:

(1) $$\mathrm{BP}=-2\sigma (\frac{\cos\theta_{I}^{*}}{w}+\frac{\cos\theta_{A}}{h})$$

where *σ* is the surface tension of the liquid, $\theta_{I}^{*}$ is the minimum value between $\theta_{A}+\beta$ and 180°, where *β* is the outlet angle and $\theta_{A}$ is the contact angle of the liquid on the channel, and *w* and *h* are the width and height of the channel, respectively. When the fluid pressure exceeds the BP, the CBV ruptures. Choi et al. [90] designed a colorimetric sweat sensor with multiple reaction zones based on CBVs (Figure 4a). Sweat enters the reaction zone through the inlet and successively passes through CBVs #1 and CBVs #2, filling chambers 1 and 2 with sweat, then CBVs #3 passes through, and sweat flows to the next reaction zone. When all the reaction zones are completely filled, CBVs #4 passes through, and waste is discharged from the microfluidic device. The combination of multiple CBVs enables the simultaneous, independent, and real-time evaluation of multiple biomarkers by a single device. Similarly, Zhang et al. [91] used CBVs with two different BPs to achieve sequential sweat sampling (Figure 4b). The BP of CBVs #1 is smaller than that of CBVs #2. Sweat enters the device through the inlet, filling three circular detection chambers successively. As sweat is continuously pumped in, the pressure gradually exceeds the BP of CBVs #2, and CBVs #2 passes through, discharging waste.

A non-electrochemical sweat sensor based on microfluidic valves utilizes SAP with high expansion coefficients. When a suitable amount of polymer is placed in a microfluidic chamber, it forms a polymer valve. When liquid enters the chamber, the polymer expands upon contact with water, filling the microfluidic chamber and blocking the fluid flow path. Kim et al. [92] proposed a microfluidic device for measuring the concentration of ammonia and ethanol in sweat based on SAP (Figure 4c). When sweat enters the SAP chamber, the polymer absorbs water and expands, pushing the solution above into the detection zone while blocking the microfluidic channel to prevent backflow of the sample and contamination. Similarly, Kim et al. [93] proposed a microfluidic device that combines SAP and hydrophobic valves to measure sweat rate and chloride (Figure 4d). Hydrophilic channels with positive capillary force draw sweat into channel 1, but the presence of a hydrophobic valve directs the sweat to channel 2, where the detection zone is located. After filling the detection zone, sweat continues to flow to the SAP, where the polymer expands to block the fluid path, and the sweat flows into channel 3.

Microchannels in microfluidic devices are on the micrometer scale. Hydrophilic channels have a positive capillary force that draws sweat into the microchannels, while hydrophobic channels have a negative capillary force that suppresses liquid entry [94,95,96]. Partial hydrophobic modification in the hydrophilic channel forms a hydrophobic valve that can control the liquid flow direction. Zhang et al. [97] proposed a microfluidic device for timed sampling of sweat using hydrophobic valves (Figure 4e). The device is made of PDMS and has three collection chambers (T1, T2, and T3). PDMS has natural hydrophobicity and is partially modified with hydrophilic treatment in some microchannels and collection chambers. The unmodified part of the microchannel remains hydrophobic, forming a hydrophobic valve. When sweat glands secrete sweat, the positive capillary force draws sweat into the microchannel. The hydrophobic valve causes the sweat to flow along the chamber wall and fill the collection chamber. As sweat continues to be secreted, the hydrophobic valve opens, and the sweat flows to the next chamber.

Capillary valves are based on the tension of water, which can change the width, height, and exit angle of channels to create valves with different BPs. SAP valves rely on the swelling properties of the polymer to block microfluidic channels, forcing a change in the direction of the fluid flow. Hydrophobic valves use capillary force to promote liquid flow in hydrophilic walls and prevent flow in hydrophobic walls, causing liquid to flow along the hydrophilic wall. The use of valve structures can guide sweat into the microchannels for timed sampling, avoid liquid mixing, and achieve accurate and timed analysis of sweat.

## 3. Testing Methods

### 3.1. Colorimetric Methods

The colorimetric method relies on the chemical reaction or interaction between the reagent and the analyte to cause a color change in the reagent, which results in a quantifiable change in optical wavelength or intensity [98,99,100]. This method is typically semi-quantitatively read with the naked eye or quantitatively read with color extraction and smartphones or digital cameras.

**Figure 5:**
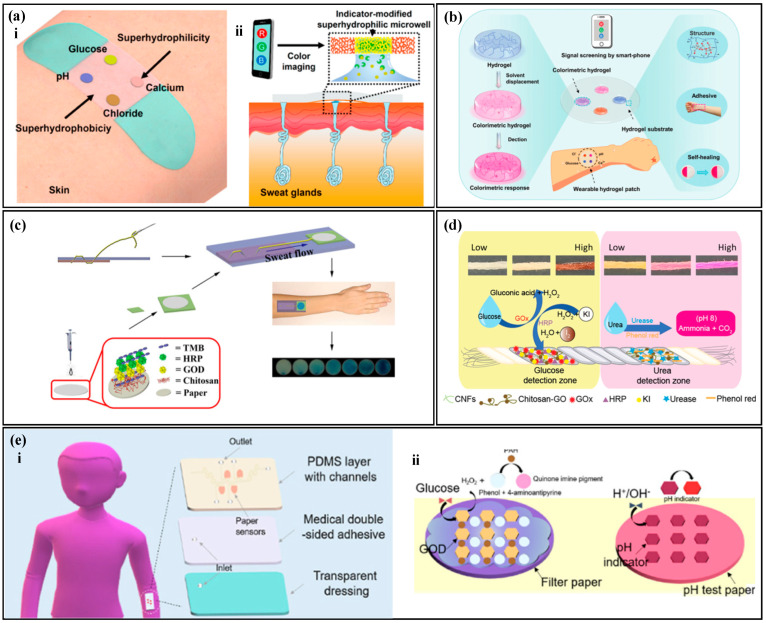
Colorimetric methods. (**a**) (i) Schematic diagram of a colorimetric biosensor with a hydrophilic and hydrophobic microarray. (ii) Cross-sectional diagram of a colorimetric biosensor with a hydrophilic and hydrophobic microarray. Reproduced with permission [104]. Copyright 2019. Reproduced with permission from the American Chemical Society. (**b**) Water gel colorimetric sensor with self-healing properties. Reproduced with permission [105]. Copyright 2021. Reproduced with permission from the Royal Society of Chemistry. (**c**) Thread-based glucose colorimetric sensor. Reproduced with permission [36]. Copyright 2019. Reproduced with permission from the Springer Netherlands. (**d**) Cotton thread colorimetric sensor for measuring sweat glucose and urea. Reproduced with permission [106]. Copyright 2020. Reproduced with permission from Elsevier. (**e**) (i) Schematic diagram of colorimetric sweat sensor for measuring glucose and pH based on a Tesla valve. (ii) Detection principle of the colorimetric sweat sensor for measuring glucose and pH based on a Tesla valve. Reproduced with permission [107]. Copyright 2022. Reproduced with permission from Elsevier.

The concentration of the analyte is correlated with the color of the reagent, and by measuring the optical information of the reagent, the concentration of the analyte can be analyzed [101,102,103]. Typically, the analysis of inorganic substances, such as chloride ions, calcium ions, and hydrogen ions, is relatively simple and the reactions with colorimetric reagents are reversible. He et al. [104] proposed a colorimetric sensor with a hydrophobic-hydrophilic microarray (Figure 5a) for the detection of chloride ions, hydrogen ions, calcium ions, and glucose in human sweat. They used a mercury ion-containing reagent to detect chloride ions in sweat, where the color of the reagent changed from white to yellow with an increase in chloride ion concentration. The hydrogen ion concentration was tested using a universal pH indicator, where the pH value increased and the color changed from red to purple. The calcium ion concentration was measured using theo-cresolphthalein complexone method, where the degree of purple of the reagent increased as the concentration of the target analyte increased. Similarly, Wang et al. [105] combined the reagent with a hydrogel to prepare a self-healing hydrogel colorimetric sensor (Figure 5b) for the detection of chloride ions, hydrogen ions, calcium ions, and glucose in human sweat.

The analysis of organic substances such as glucose, lactate, and urea require multiple steps involving enzyme catalysis and the generation of byproducts that react with the reagent to cause a color change. The color of the reagent is correlated with the concentration of the target analyte in sweat, and the process of enzyme-based analysis of organic substances is irreversible. Xiao et al. [36] proposed a thread-based glucose colorimetric sensor (Figure 5c), The colorimetric area was coated with glucose oxidase (GOx), peroxidase, and a colorimetric dye. When sweat flow into the colorimetric region, glucose in sweat reacted with GOx to produce hydrogen peroxide, which was oxidized by peroxidase and reacted with the reagent, changing its color from light yellow to blue. The color change in the colorimetric area was captured by a smartphone for the analysis of the target analyte concentration. Promphet et al. [106] proposed a cotton thread colorimetric sensor (Figure 5d) for the measurement of glucose and urea in sweat. The detection area with urease and phenol red (a pH indicator dye) to detect urea. Urea in sweat reacted with urease to produce ammonia and carbon dioxide, which increased the local pH and caused the phenol red to change color. Similarly, Shi et al. [107] used a Tesla valve for timed sampling of sweat (Figure 5e) to measure glucose and pH values in sweat.

In colorimetric sweat sensors, biomarkers in sweat react with specific colorimetric reagents, leading to a modification in the colorimetric reagent’s color information, which is measured to analyze the concentration of a designated biomarkers in sweat. Typically, the measurement steps for inorganic substances in sweat are relatively straightforward, and the process of biomarker interaction with colorimetric reagents is reversible. Changes in the concentration of the target substance result in a transition of the colorimetric reagent between two colors. Conversely, the measurement steps for organic substances are more complex. Enzymes are employed to enzymatically decompose the organic compounds, generating secondary reaction products. These secondary reaction products subsequently react with the colorimetric reagent, causing a change in its color. Due to the decomposition of organic compounds and the oxidation of the generated secondary reaction products during this process, it is considered an irreversible reaction. Additionally, the consumption of enzymes poses challenges to the longevity of this type of sensor.

### 3.2. Fluorescence Methods

The accuracy of detecting sweat biomarkers using colorimetric methods is relatively limited in terms of both precision and detection range. As a result, the effectiveness of these methods in accurately identifying sweat biomarkers is not particularly prominent. In contrast, fluorescence methods have the potential to significantly improve the detection sensitivity of sweat biomarkers. This enhancement enables the detection of lower concentrations of biomarkers present in sweat samples. Furthermore, fluorescence methods offer the advantage of faster detection speeds, allowing for more efficient and timely analysis of sweat biomarkers [108,109,110]. Under specific light source irradiation, the fluorescence probe reacts with the biomarker, causing a change in the excited fluorescence intensity. The fluorescence intensity change is detected using a smartphone to calculate the target molecule concentration [111,112].

Under specific light source irradiation, some substances are in an excited state and emit fluorescence that reflects the characteristics of the reactants. Quantitative analysis of substances can be performed by analyzing the fluorescence [113,114]. As some substances do not emit fluorescence themselves (or have weak fluorescence), it is necessary to convert them into substances that can emit fluorescence [115,116]. For example, some reagents (such as fluorescent dyes) can be used to form complexes with substances that do not emit fluorescence, and the complexes can emit fluorescence for measurement.

**Figure 6:**
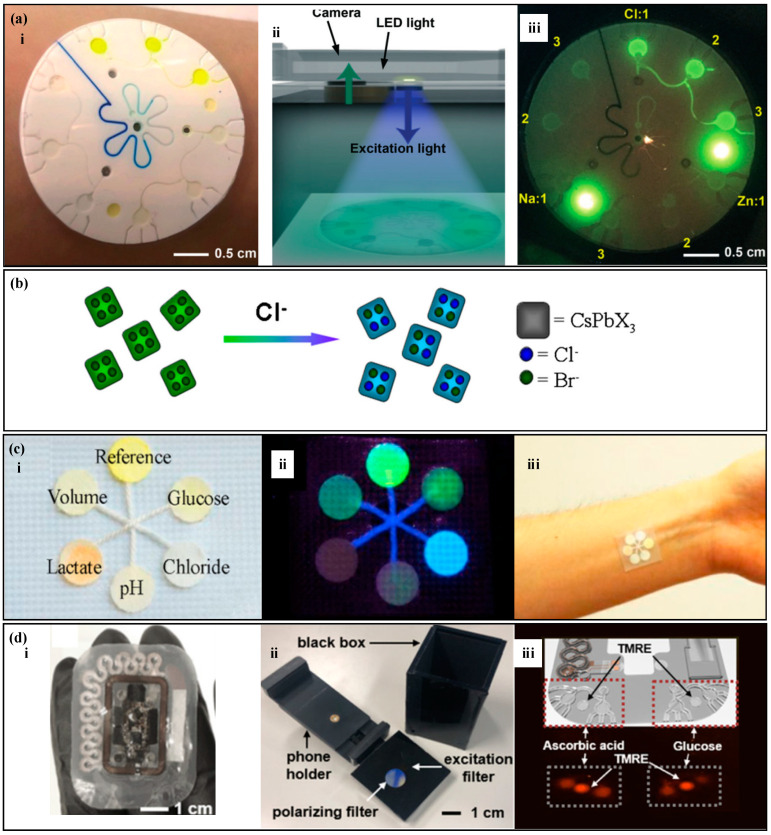
Fluorescence methods. (**a**) (i) A fluorescence sweat sensor with a bursting valve. (ii) Signal detection device, including a smartphone and a color filter. (iii) Blue light image captured by the smartphone. Reproduced with permission [117]. Copyright 2018. Reproduced with permission from the Royal Society of Chemistry. (**b**) Schematic diagram of the exchange between fluorescence probe and chloride ions. Reproduced with permission [74]. Copyright 2021. Reproduced with permission from the Royal Society of Chemistry. (**c**) (i) A thread-based fluorescence sweat sensor. (ii) Sensing image under ultraviolet light. (iii) The thread-based fluorescence sweat sensor worn on the forearm. Reproduced with permission [80]. Copyright 2020. Reproduced with permission from Elsevier. (**d**) (i) A fluorescence sensor with a skeletal microfluidic network. (ii) Device used for fluorescence reading. (iii) Fluorescence images of ascorbic acid, glucose signals and reference (TMRE) signals. Reproduced with permission [122]. Copyright 2021. Reproduced with permission from Springer India.

Analysis of inorganic substances in fluorescence methods is relatively simple. The fluorescence probe in the detection area reacts with the target substance and, under the irradiation of a specific light source, the mixture in the detection area emits light. The target substance concentration is correlated with the fluorescence intensity. Sekine et al. [117] proposed a fluorescence sweat sensor with a burst valve (Figure 6a) to analyze the concentration of sodium ions, chloride ions, and zinc ions in sweat. The device has three partitions, with four reaction zones in each partition. Using a burst valve to time the sampling, the corresponding fluorescence probe is placed in each reaction zone. Under the irradiation of a specific wavelength of light, the fluorescence probe in the reaction zone reacts with the target substance and emits light. The fluorescence intensity is measured using a smartphone to analyze the target substance concentration. Similarly, Li et al. [74] used fluorescence methods to measure the concentration of chloride ions in sweat (Figure 6b). Under the irradiation of ultraviolet light, the fluorescent agent and chloride ions undergo rapid halogen exchange, resulting in an obvious blue shift in wavelength and a change in fluorescence color from green to blue, achieving rapid detection of chloride ions in sweat.

There are similarities and differences between fluorescence measurement and colorimetric methods for the measurement of organic compounds. For example, glucose and ascorbic acid are measured using enzyme–substrate reactions that generate hydrogen peroxide as a byproduct, which reacts with fluorescent probes to produce fluorescence [118,119]. However, in fluorescence sweat sensors, the measurement of lactate does not necessarily require the involvement of enzymatic substances [120,121]. Lactate forms a complex with fluorescein/Fe(III), and the fluorescence intensity of the complex is used to analyze the concentration of the target substance. Ardalan et al. [80] developed a thread-based fluorescence sweat sensor (Figure 6c), which measures glucose, lactate, pH, chloride ions, and sweat rate. Sweat is collected on threads and transported to a paper-based fluorescent probe. The glucose detection area is coated with GOx, horseradish peroxidase (HRP), and a fluorescent probe to detect hydrogen peroxide as a byproduct, enabling glucose detection. The lactate detection area is coated with fluorescein/Fe(III), and the fluorescence intensity of the complex is used to quantitatively analyze lactate concentration. Kim et al. [122] proposed a fluorescent sensor with a skeletal microfluidic network (Figure 6d), which is used to measure ascorbic acid and glucose. A burst valve is used for timed sampling of sweat. The reaction area is equipped with corresponding oxidases, peroxidases, and fluorescent probes. After the sweat enters and reacts in the reaction area, the device is placed in a box with a light source emission function and analyzed using a smartphone to determine the concentration of the target substance based on its fluorescence intensity.

Fluorescence measurement is based on photo-induced luminescence, where specific light sources excite molecules to undergo energy level transitions and emit light. The fluorescence intensity has a correlation with the concentration of the target substance, and the concentration can be analyzed by measuring the fluorescence intensity. Fluorescence measurement is fast, highly sensitive, and can quickly analyze trace biomarkers in the human body, achieving rapid quantitative analysis of biological marker concentrations.

### 3.3. Other Detection Methods

With the development of sweat sensors, many methods have emerged for the quantitative analysis of sweat biomarkers, such as SERS and visual semi-quantitative reading.

**Figure 7:**
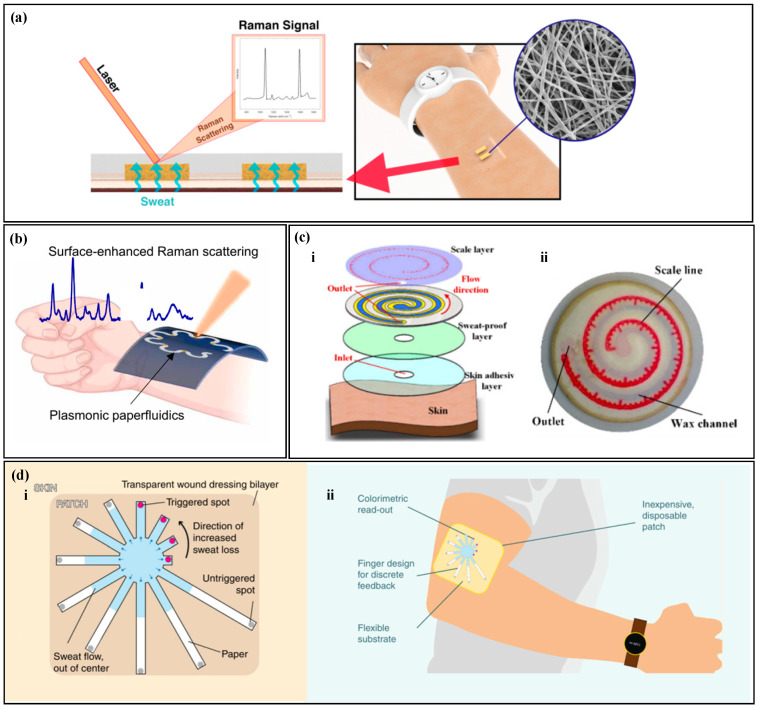
Other Detection Methods. (**a**) A pH sweat sensor based on surface-enhanced Raman scattering (SERS). Reproduced with permission [130]. Copyright 2021. Reproduced with permission from the American Chemical Society. (**b**) A paper-based serpentine microfluidic device based on SERS. Reproduced with permission [68]. Copyright 2022. Reproduced with permission from the American Association for the Advancement of Science. (**c**) (i) An exploded view of a paper-based microfluidic chip with semi-quantitative visual readout. (ii) A photo of the paper-based microfluidic chip with semi-quantitative visual readout. Reproduced with permission [132]. Copyright 2022. Reproduced with permission from the MDPI. (**d**) (i) A paper-based sweat sensor with a discrete colorimetric indicator. (ii) A schematic of the wearable device. Reproduced with permission [133]. Copyright 2019. Reproduced with permission from the Springer Nature.

SERS is a highly sensitive analytical method that can detect and quantify various analytes, including metabolites, macromolecules, and microorganisms [123,124]. Raman scattering refers to the occurrence of a specific frequency shift in the scattered spectrum when incident light interacts with surface molecules, and this shift can be measured to investigate information about molecular structure and composition [125,126]. However, the signals emitted by Raman scattering are very weak, plasma nanostructures can greatly enhance the Raman scattering of analytes near the surface of the nanostructure, known as the surface-enhanced Raman scattering effect, and the corresponding spectrum is called SERS [127,128]. Surface-enhanced Raman spectroscopy has high sensitivity and stability, and the intensity of the spectrum is proportional to the sample concentration, making it possible to quantitatively analyze target substances at very low concentrations [129]. Chung et al. proposed a pH sweat sensor based on SERS (Figure 7a) [130]; as a substrate for Surface-Enhanced Raman Scattering (SERS), thermoplastic polyurethane (TPU) films are used to rapidly and accurately analyze the pH of sweat under laser illumination. Similarly, Mogera et al. designed a paper-based serpentine microfluidic device (Figure 7b), Ref. [68] using SERS to measure sweat rate and uric acid in sweat. The design of the serpentine channel allows the device to adapt to skin deformation, and circular SERS substrates modified by nanogold are set at different positions in the serpentine channel to quantify the concentration of sweat analytes at different time points. In addition, there is a significant color contrast between the wet and dry areas, and the sweat rate is quantified based on the distance of liquid advancement. It is worth noting that Localized Surface Plasmon Resonance (LSPR) technology is also a highly sensitive detection method. When molecules in sweat interact with the surface of metal nanoparticles, it leads to changes in the dielectric constant, thereby affecting the plasmon resonance frequency. The analysis of specific components in sweat is achieved by monitoring the changes in resonance frequency. For instance, Nan et al. have proposed a flexible LSPR biosensor where cortisol aptamers are immobilized on the surface of the substrate, enabling highly sensitive and selective detection of cortisol [131].

Colorimetry, fluorescence, and SERS require external electronic devices for reading, which still have limitations. Semi-quantitative reading with the naked eye does not require external equipment and can achieve fast readings. Wang et al. proposed a paper-based microfluidic chip for measuring sweat rate (Figure 7c) [132], which uses wax printing technology to construct microchannels on filter paper. Cobalt (II) chloride without water is modified in the microchannel, and a tape with a scale is placed on the top of the filter paper. When sweat enters the microfluidic device, cobalt (II) chloride without water changes from blue to red, and the amount of sweating can be directly read from the scale on the tape. Jain et al. [133] proposed a paper-based microfluidic device with a discrete colorimetric indicator (Figure 7d) for measuring sweat rate. Radially arranged filter paper is used as microfluidic channels, and a dye is set at the end of each channel. When the sweat completely fills the microchannel, the dye at the end changes color. By calculating the duration of sweat entering the channel until it reaches the colorimetric immersion zone, linear discretization of visual readings is achieved.

Traditional colorimetry and fluorescence often rely on enzyme and antibody analysis of biomarkers, but enzymes and antibodies can degrade over time or become ineffective when exposed to harsh environments. SERS can perform highly sensitive quantitative analysis of target substances without the involvement of enzymes and antibodies. Devices with scales or indicator marks can achieve semi-quantitative reading with the naked eye. Semi-quantitative reading with the naked eye does not require complex external equipment, and physiological parameters of the human body can be read in real-time. Such devices are easy to manufacture, cost-effective, and can be produced on a large scale.

## 4. Signal Reading Methods

### 4.1. Optical Three-Primary-Color (RGB) Method

The color change of the colorimetric reagent is correlated with the concentration of the target substance, but precise readings are difficult to achieve with the naked eye. It is necessary to convert the color into an accurate physical quantity by analyzing the image with electronic devices for precise readings [124,125]. Any color that the naked eye can see in nature can be mixed and superimposed from the optical three-primary colors, which refer to red (R), green (G), and blue (B). By analyzing the RGB values of the image, the concentration of the target substance can be quantitatively analyzed [134,135].

**Figure 8:**
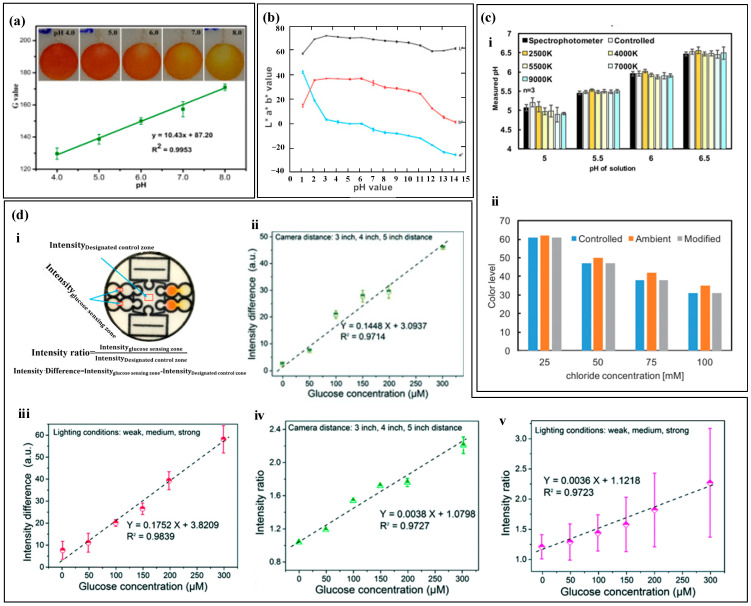
Optical three-primary-color (RGB) method. (**a**) The relationship between the G value and pH value of artificial sweat. Reproduced with permission [82]. Copyright 2020. Reproduced with permission from the Springer Nature. (**b**) The segmented calibration curve of RGB values. Reproduced with permission [29]. Copyright 2019. Reproduced with permission from Elsevier. (**c**) (i) The measurement results of pH value under different lighting intensities. (ii) The measurement results under controlled lighting conditions with a standard color card as reference. Reproduced with permission [90]. Copyright 2019. Reproduced with permission from the Ameri-can Chemical Society. (**d**) (i) Schematic diagram and calculation formula of the specified control area. (ii) The average glucose standard curve determined by intensity difference at 3 inches, 4 inches, and 5 inches distance. (iii) The average blood glucose standard curve determined by intensity difference under weak, medium, and strong light conditions. (iv) The average glucose standard curve determined by intensity ratio at 3 inches, 4 inches, and 5 inches distance. (v) The average blood glucose standard curve determined by intensity ratio under weak, medium, and strong light conditions. Reproduced with permission [76]. Copyright 2019. Reproduced with permission from the Royal Society of Chemistry.

After the colorimetric reagent reacts with biomarkers of different concentrations, it exhibits varying shades of color changes. By capturing images of the reaction products using a smartphone and converting them into digital RGB data, an RGB-to-biomarker concentration calibration equation can be established [135,136]. For example, Xiao et al. [82] used colorimetry to measure sweat pH (Figure 8a). The pH value ranged from 4.0 to 8.0, and the color changed from orange to yellow. They used a smartphone to extract the single R, G, and B values of the detection area. Among them, the G value had a linear relationship with sweat pH, and the color difference was the most significant, which was used to quantify the concentration of the target substance. Promphet et al. [29] segmented the calibration curve of RGB values (Figure 8b). The intercept slopes of each segment calibration curve were different and corresponded to a concentration range. Based on the slope changes, the calibration curve was divided into seven regions to improve the accuracy of target substance detection.

The absolute color extracted from digital images using colorimetric analysis depends on the lighting conditions [90], as shown in Figure 8c, which includes four lighting environments Colorimetric analysis results may have certain errors due to lighting conditions, and also due to dust, debris, emulsion or other substances that may contaminate the top of the device and cause color extraction errors. In order to improve accuracy and reduce errors caused by environmental light sources, black and white reference colors and standard reference colors were set up for color reading calibration before RGB value collection. The RGB values of the reference marker and the detection point were recognized using a smartphone, and the color information of the reference marker was used to calibrate the RGB values of the detection point. In addition to lighting conditions, the distance between the camera and the detection area may also affect the measurement results of colorimetric analysis. Zhang et al. [76] used intensity difference and intensity ratio to calibrate the measurement results of glucose (Figure 8d). The study showed that the shooting distance and exposure intensity can seriously affect the measurement results of the sensor, and the standard curve based on the intensity difference and intensity ratio has a linear relationship with glucose concentration, greatly reducing the measurement error of the target material.

RGB values are the three primary colors of light, and their numerical values are correlated with the concentration of the target material. Analyzing RGB values can quantify the concentration of the target material. However, external environmental factors such as lighting intensity, lighting color, and shooting distance may cause deviations in the extracted color information. Therefore, introducing standard colors as reference colors can calibrate the color of the detected area to improve the detection accuracy, or perform subtraction or division operations on color data from different areas to reduce errors.

### 4.2. Chromaticity Diagram Method

In colorimetric sweat sensors, the optical image of the colorimetric reagent is captured, and color is quantified to quantitatively assess the levels of the target substance The concentration of the target substance is not only directly related to a single R, G, or B value, but often requires comprehensive consideration [137,138]. The RGB values are converted into two-dimensional chromaticity coordinates (x, y) in the color space. Any point on the two-dimensional chromaticity diagram can represent the information of the three RGB values [139,140].

**Figure 9:**
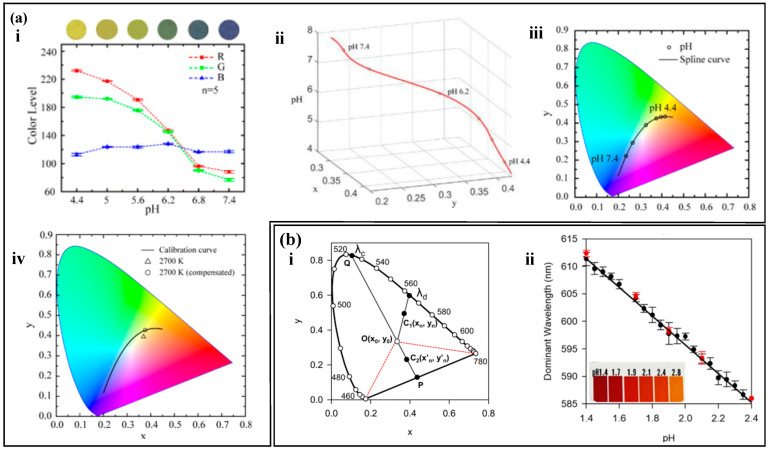
Chromaticity Diagram Method. (**a**) (i) Relationship between RGB values and the concentration of the analyte. (ii) Three-dimensional calibration curve of pH values established by spline interpolation. (iii) Standard pH calibration curve in the CIE 1931 color space. (iv) pH calibration curve obtained under different lighting conditions on the chromaticity diagram. Reproduced with permission [62]. Copyright 2021. Reproduced with permission from the American Chemical Society. (**b**) (i) Main wavelength (*λ*_d_) and complementary wavelength determined on the CIE 1931 diagram. (ii) pH calibration curve determined using the main wavelength. Reproduced with permission [152]. Copyright 2021. Reproduced with permission from the Analytical Chemistry.

The RGB color model can be used to represent colors by creating a three-dimensional coordinate system. The Red, Green, and Blue values are used to represent the X, Y, and Z axes, respectively, and a cubic space is constructed using additive color mixing to form a color space based on the RGB model [141,142]. Any point in this space has a unique RGB value. However, the RGB model is three-dimensional, so theoretical RGB primaries are derived through mathematical methods based on the RGB model. A two-dimensional standard chromaticity system is created using the processed r-axis and g-axis to represent all colors except black [143,144,145]. Not all target substance concentrations are only related to a single value in the RGB model, and often require comprehensive consideration. For example, pH values are related to all three values in the RGB model (Figure 9a (i)). The pH concentration is represented by chromaticity coordinates, and a series of standard 3D calibration curves for the analysis of substances are established using spline interpolation (Figure 9a (ii)). The substance is further quantified, and the three-dimensional calibration curve is mapped onto a two-dimensional chromaticity diagram. Any point on the chromaticity diagram calibration curve is associated with the concentration of the target substance and the three RGB values (Figure 9a (iii)). The chromaticity characteristics of colorimetric analysis are largely influenced by the spectral characteristics of the light source and the RGB responsiveness of the camera. For example, the concentration of the target substance read from the chromaticity diagram varies with different color temperatures. The introduction of compensation functions can calibrate the measurement results (Figure 9a (iv)).

Any color can be seen as a mixture of a certain spectral color and a reference light source in a certain proportion. This spectral color is the main wavelength ($\lambda_{d}$) of the color [146]. On the CIE chromaticity diagram, the chromaticity points (i.e., chromaticity coordinates) of the color sample and the light source are marked, and a straight line is drawn connecting the two points. The line is extended from the light source towards the chromaticity point of the sample, and where it intersects with the spectral locus is the wavelength of the dominant wavelength of the color sample [147,148,149]. In colorimetric analysis, the change in the color of the color reagent often accompanies a significant change in hue, and the most relevant parameter for color tone is the main wavelength (or complementary wavelength) [150,151]. Analyzing the main wavelength (or complementary wavelength) in the color information of the color reagent can be used to analyze the concentration of the target substance. Li et al. [152] analyzed the pH value using the main wavelength and complementary wavelength (Figure 9b). They obtained the RGB values of the image using a self-developed mobile app, linearized and transformed them into chromaticity coordinates, and solved the main wavelength (or complementary wavelength) through the chromaticity coordinates. The main wavelength (or complementary wavelength) is related to the pH value, achieving high-precision measurement of pH value.

The color information of the target substance is complex, and a single RGB value is insufficient to represent the concentration of the target substance. The coordinates of a point on the two-dimensional chromaticity coordinates can represent the three RGB values, and the target substance’s calibration curve can be fitted using the two-dimensional chromaticity coordinates to quantify the concentration of the target substance. In addition, environmental factors, such as lighting, can affect the extraction of RGB values. Introducing a calibration curve can calibrate the concentration of the target substance. Color changes often accompany significant changes in hue, and the most relevant parameter for hue is the main wavelength (or complementary wavelength). Using the main wavelength to quantify the concentration of the target substance achieves precise measurement of the target substance.

### 4.3. Spectrum Analysis and Calibration Method

**Figure 10:**
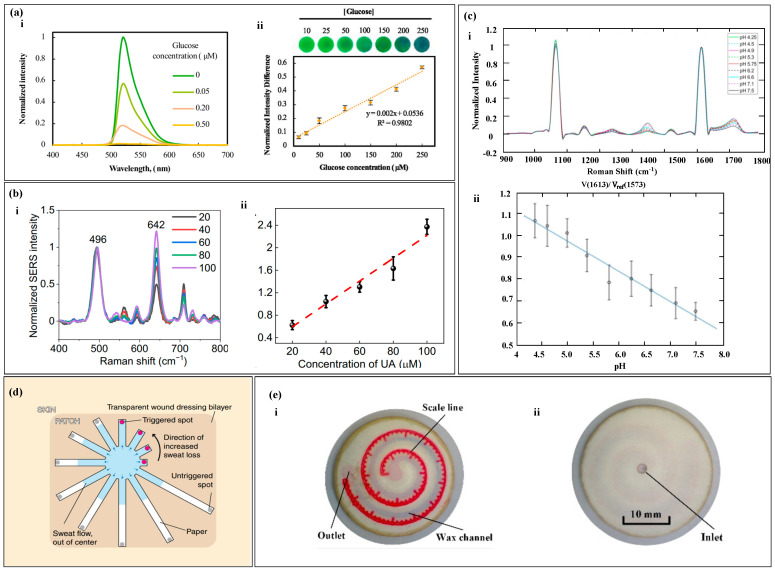
Spectrum Analysis and Calibration Method. (**a**) (i) shows the fluorescence spectra of the glucose probe in artificial sweat of varying glucose concentrations under the condition of λ excitation wavelength of 365 nm. (ii) The calibration curve of glucose fluorescence intensity is shown. Reproduced with permission [80]. Copyright 2020. Reproduced with permission from Elsevier. (**b**) (i) displays the surface-enhanced Raman spectra of uric acid at different concentrations. (ii) The calibration curve of the SERS intensity ratio for uric acid quantification is shown. Reproduced with permission [68]. Copyright 2022. Reproduced with permission from the American Association for the Advancement of Science. (**c**) (i) Presentation of the SERS spectra on nanofibers within the pH range of sweat. (ii) The pH calibration curve based on SERS is shown. Reproduced with permission [130]. Copyright 2021. Reproduced with permission from the American Chemical Society. (**d**) Depiction of a paper-based sweat sensor with discrete colorimetric markers. Reproduced with permission [132]. Copyright 2022. Reproduced with permission from the MDPI. (**e**) (i,ii) The front and back of a paper-based sweat rate monitoring chip with indicator scales. Reproduced with permission [133]. Copyright 2019. Reproduced with permission from the Springer Nature.

The RGB and colorimetric methods analyze the concentration of a target substance by examining changes in the color of the reactants. However, in sweat samples, the presence of other biomarkers that can react with the color reagents can reduce the accuracy of the analysis. Conversely, the fluorescence method involves analyzing the fluorescence spectrum of the target molecules. When exposed to specific wavelengths, only the target molecules emit a fluorescence signal. Similarly, through appropriate surface modifications, the SERS substrate surface can exclusively generate SERS signals from the target molecules. Consequently, the analysis of fluorescence spectra and SERS can enhance the accuracy of the results. Additionally, in the absence of electronic devices, semi-quantitative readings can be achieved by visual observation. Fluorescence quenching refers to the interaction between fluorescent molecules and solvent molecules or other solute molecules, leading to a decrease in fluorescence intensity [153,154,155]. In fluorescence measurement and analysis, the quenching of the fluorescent agent due to the reaction with the target substance is correlated with the concentration of the target substance [156,157]. By measuring the difference in the fluorescent intensity of the fluorescent agent, the concentration of the target substance can be analyzed [158,159]. For example, in the determination of glucose, under the action of peroxidase, fluorescein is oxidized and quenched, and the degree of quenching is correlated with the glucose concentration. By measuring the fluorescence spectra of different glucose concentrations and normalizing the fluorescent difference, the glucose concentration is proportional to the normalized fluorescent difference (Figure 10a) [80]. The phenomenon where the frequency or wavelength of light changes upon illumination on a substance is known as Raman scattering. The use of a substrate with plasmonic nanostructures can enhance Raman scattering, leading to what is called Surface-Enhanced Raman Spectrum (SERS). The intensity of SERS spectra correlates with the concentration of the target substance in the sample, where the intensity of the spectrum has a correlation with the sample concentration. By processing the difference in the intensity of the spectrum, the processed spectrum intensity is proportional to the concentration of the target substance (Figure 10b,c) [68,130].

Colorimetry and spectroscopic analysis require additional electronic devices for data reading, and the data reading steps are cumbersome and difficult to meet people’s daily use, especially in harsh environments such as sports events and construction sites, where real-time reading is difficult. For example, in a marathon, the tester is in a state of motion and needs to remove the sensor and use an instrument for analysis to read the data, making it impossible to read in real-time. The human eye is a natural camera, and semi-quantitative reading can be done using the naked eye. By designing the sensor into a regular shape and setting relevant scales or reference points (Figure 10d,e), sweat will flow along the designed fluid channel, and the fluid flow can be observed with the naked eye. Reading can be quickly achieved by referring to the reference points integrated in the sensor [132,133].

Fluorescence spectra and SERS are methods that analyze spectral information, such as wavelength and peak values, to achieve quantitative analysis of sweat target substances. These methods have high sensitivity and accuracy, but the analysis steps are cumbersome and require additional measuring equipment. The human eye is a natural camera, and semi-quantitative reading can be done using the naked eye. Microfluidic devices for naked eye reading have the advantages of low cost and simple production and can be applied in a variety of scenarios without the need for electronic devices to achieve real-time reading, which can meet the needs of most people in daily life.

## 5. Applications

Sweat is secreted by sweat glands and transported to the epidermis through dermal ducts. During the process of sweat secretion, biomarkers such as metabolites, electrolytes, and trace elements from surrounding cells and interstitial fluid are absorbed into the sweat [82,160,161], so sweat contains abundant biomarkers that can provide rich information related to physiological states. In recent years, there has been a surge in the adoption of non-electrochemical methods for detecting biomarkers in human sweat, as illustrated in Table 2.

**Figure 11:**
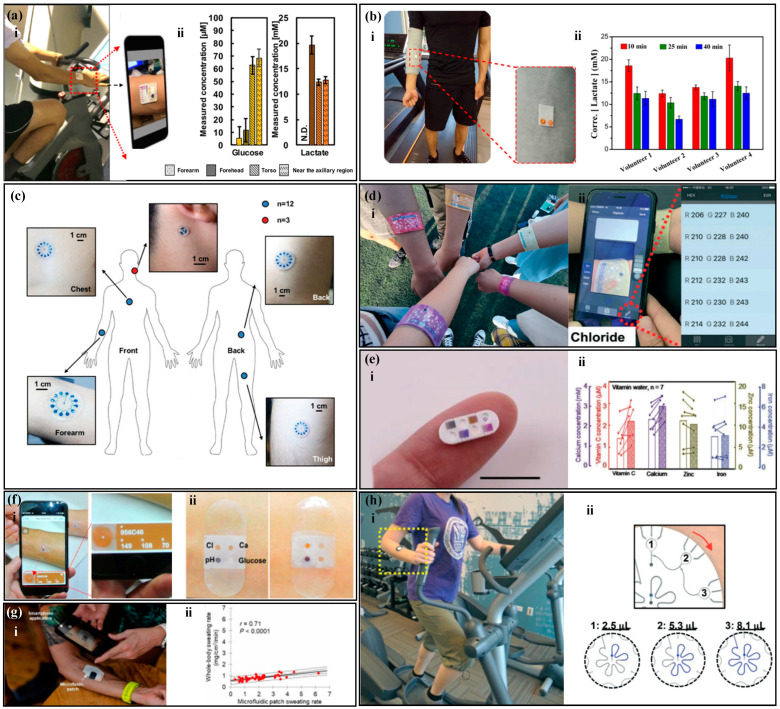
Applications of non-electrochemical sweat sensors. (**a**) (i) A CBVs-based sweat sensor with the function of measuring human glucose, lactate, and chloride. (ii) Sweat glucose and lactate concentrations in different positions on the subject’s forearm, forehead, chest, and armpit. Reproduced with permission [90]. Copyright 2019. Reproduced with permission from the Ameri-can Chemical Society. (**b**) (i) A thread-based sweat sensor for measuring pH and lactate. (ii) Sweat pH and lactate concentrations after 10, 25, and 40 min of running on the subject. Reproduced with permission [82]. Copyright 2020. Reproduced with permission from the Springer Nature. (**c**) A CBV-based sweat sensor with the function of measuring human potassium, sodium ions, and lactate. Reproduced with permission [175]. Copyright 2017. Reproduced with permission from the John Wiley and Sons Ltd. (**d**) (i) A thread-based sweat sensor for measuring chloride. (ii) A smartphone application captures the image of the target area to convert into RGB values. Reproduced with permission [81]. Copyright 2021. Reproduced with permission from the Royal Society of Chemistry. (**e**) (i) A colorimetric device for monitoring and delivering vitamin C, calcium, zinc, and iron. (ii) Concentration changes of vitamin C, calcium, zinc, and iron in blood and sweat before and after drinking orange juice. Reproduced with permission [193]. Copyright 2022. Reproduced with permission from the Wiley–VCH Verlag. (**f**) (i) A patch-type colorimetric sweat sensor with the function of measuring calcium ions, pH, glucose, and chloride ions. (ii) Color contrast before (left) and after (right) sweat secretion. Reproduced with permission [104]. Copyright 2019. Reproduced with permission from the American Chemical Society. (**g**) (i) A flexible sweat sensor for measuring sweat rate and chloride ion concentration. (ii) Local chloride sweat concentration with microfluidic control compared to whole-body chloride sweat concentration. Reproduced with permission [201]. Copyright 2020. Reproduced with permission from the American Association for the Advancement of Science. (**h**) (i) A CBVs-based sweat sensor with the function of measuring human sweat rate. (ii) A schematic diagram of sweat flow. Reproduced with permission [117]. Copyright 2018. Reproduced with permission from the Royal Society of Chemistry.

There are various types of sweat metabolites, such as lactate, glucose, and cortisol [162,163]. Studies have shown that there is a correlation between glucose concentration in sweat and blood glucose concentration, and the concentration of glucose in sweat is about 1% of that in plasma [164,165]. Based on the concentration of glucose in sweat, the physical condition of diabetes patients can be diagnosed and monitored. Lactate is a direct product of anaerobic metabolism, and evaluating its content can reflect the intensity of physical exercise and provide a warning signal for pressure ischemia [122,166,167]. Chio et al. [90] developed a sweat sensor based on CBVs (Figure 11a), which can perform timed sampling and measurement of glucose, lactate, and chloride in human sweat. The experimental results showed that the glucose concentration range in the sweat of nine subjects was about 100 times lower than the normal blood glucose level, and the lactate level in the forehead area of the human body was significantly higher than that in the chest. Xiao et al. [82] proposed a thread-based sweat sensor for measuring pH and lactate (Figure 11b). When testing on the human body, the pH value was used to calibrate the lactate value. The experiment found that sweating during exercise diluted lactate in human sweat, leading to a decreasing trend in lactate concentration in all volunteers. Zhang et al. [76] proposed a paper-based sweat sensor for measuring pH and glucose. The sensor set a sweating reference area to remind the tester to read the results at the appropriate time and avoid detection errors caused by reading too early or too late. Adrenal gland releases cortisol in response to body stress [168], and there is a correlation between cortisol level and stress. Long-term elevation of cortisol levels is associated with diseases, such as obesity, depression, hypertension, and diabetes [169]. Weng et al. [78] proposed a paper-based fluorescence sensor for measuring cortisol, which was worn on the surface of the human body for testing. The experimental results showed a high correlation between the detection results of the sensor and those of ELISA.

The electrolyte content in sweat can reflect the body’s water and electrolyte balance [170,171]. Potassium and sodium ions are the main electrolytes in sweat and play an important regulatory role in the balanced distribution of body fluids [172,173]. The lack of sodium ions can lead to hyponatremia, which can cause symptoms such as limb spasms, headaches, nausea, and vomiting [168]. The lack of potassium ions can lead to hypokalemia, and patients may experience symptoms such as limb weakness, mental fatigue, delayed reaction, and even heart failure [174]. Choi et al. [175] proposed a sweat sensor for measuring potassium, sodium, and lactate ions (Figure 11c), which has multiple reaction chambers with different blood plasma back pressures (BP) between them. Human tests have shown that this device can sequentially collect sweat samples at different time points and measure the concentration of potassium and sodium ions in sweat, and the measurement results are consistent with those of commercial instruments. Chloride is an important anion and has important significance in screening patients with cystic fibrosis [176]. Cystic fibrosis can seriously affect the patient’s lung function, causing malfunction of the chloride ion reabsorption channel in sweat glands, leading to abnormally high chloride ion content in the sweat of cystic fibrosis patients. Therefore, detecting chloride ions in sweat can quickly screen patients with cystic fibrosis. Zhao et al. [81] proposed a thread-based sweat sensor for measuring chloride ions (Figure 11d), in which a hydrophilic thread was sewn into a hydrophobic fabric to collect and transport sweat. Human tests have shown that the visual readings of the sensor are similar to those of the smartphone app and commercial instruments. Sweat pH has a wider range than blood pH, between 4 and 7 [176], and one of the applications of sweat pH detection can be the diagnosis of metabolic alkalosis [177]. In addition, the sweat pH of patients with dermatitis or type II kidney stones may also be abnormal. Zhang et al. [97] proposed a sweat sensor for measuring pH, which uses a hydrophobic valve for timed sampling. The device was placed on the back, neck, and forehead of the human body for testing, and the results showed that the sweat pH on the back decreased in order, but no significant difference was observed in the sweat pH on the forehead and neck. The researchers expect that this phenomenon can provide help for potential physiological changes in human body research.

Sweat contains many trace elements, such as zinc, magnesium, iron, calcium, and vitamin C [178,179]. Trace elements are essential for maintaining basic physiological functions in the human body, and their deficiency can lead to various diseases. For example, zinc deficiency is associated with immune system-related diseases such as malabsorption syndrome, chronic liver disease, chronic kidney disease, and sickle cell disease [180,181,182,183,184]. Iron is one of the essential trace elements in the human body, and its deficiency can lead to anemia and even neurodegenerative diseases [185,186]. Calcium mainly exists in the bones and teeth of the human body, and its deficiency can cause changes in teeth and osteoporosis [187,188]. Magnesium ions affect the “channels” of potassium, sodium, and calcium ions moving inside and outside cells [189,190]. Vitamin C deficiency can lead to scurvy, a disease characterized by bleeding, and abnormal formation of bone and dental tissue [191,192]. Kim et al. [193] proposed a colorimetric device for monitoring and delivering vitamin C, calcium, zinc, and iron (Figure 11e). When the user observes a low concentration of trace elements in their body, they can balance the body’s nutritional management system by releasing the nutrients in the device. Compared with taking oral medications, this device can gradually and continuously release trace elements over a long period of time. The device was worn on the human body for in vivo testing, and the concentration of vitamin C and calcium in sweat significantly increased after the tester drank orange juice, while the zinc and iron content remained unchanged, which was related to the amount of these elements in the juice. Cheng et al. [194] proposed a 3D folded paper-based colorimetric sweat sensor that uses wax printing technology to construct microfluidic channels. The device is worn on the skin to measure markers such as magnesium ions and lactate in sweat. The experiment showed that the concentration of magnesium ions in sweat significantly increased after consuming foods rich in magnesium ions. He et al. [104] proposed a band-aid-style colorimetric sweat sensor (Figure 11f) for measuring markers such as calcium ions and pH value. The device is attached to the subject’s skin, and the color of the magnesium ion reaction area changes significantly as the subject continues to exercise. The concentration of magnesium ions in sweat can be analyzed by scanning the device with a smartphone.

The human sweat rate is one of the important parameters in analyzing human sweat. Analyzing the sweat rate can evaluate the hydration status of the body [195,196]. A 2% decrease in the body’s hydration status has negative effects on the body that exceed 30% [197]. Chronic dehydration can lead to cramps, fainting, and even heat exhaustion and stroke [198,199]. In addition, the significant secretion of sweat can dilute the concentration of other target substances in sweat, so measuring the sweat rate is necessary [200]. Jain et al. [133] proposed a paper-based sweat sensor with a discrete indicator that can measure the sweat rate of the body through visual inspection. Baker et al. [201] proposed a flexible sweat sensor that measures sweat rate and chloride ion concentration (Figure 11g). The sensor can visually observe the flow of sweat in microchannels and, using a smartphone for digital image capture and analysis, it can quickly and easily evaluate the instantaneous sweat rate and sweat of microfluidic patches in a flowing environment. Human tests have shown that there is a correlation between local sweat rate and overall sweat rate, and a related algorithm was established to predict overall sweat rate. Sekine et al. [117] proposed a sweat sensor based on CBVs (Figure 11h), which can measure the sweat rate and other related information of the body. CBVs is used to connect three partitions, each of which has three miniature liquid storage tanks. The microchannels contain water-soluble dyes that produce a highly visible colored liquid front that can calculate the sweat rate of the body in real-time.

**Table 2:** Applications of the wearable sweat sensors with non-electrochemical method.

Biomarkers	Sampling	Test Method	Signal Reading	Sample Type	Ref.
glucose, pH, lactate	paper	colorimetric	RGB	human sweat	[76]
cortisol	paper	fluorescence	spectrum analysis	artificial/human sweat	[78]
pH, Cl^−^, glucose	thread	colorimetric	RGB	human sweat	[81]
lactate, pH	thread	colorimetric	RGB	artificial/human sweat	[82]
Cl^−^, pH, glucose, temperature, lactate	CBVs	colorimetric	RGB	human sweat	[90]
pH	hydrophobic valves	colorimetric	RGB	human sweat	[97]
pH, Cl^−^, glucose, Ca^2+^	paper	colorimetric	RGB	human sweat	[104]
Zn^2+^, Na^+^, Cl^−^, sweating rate	CBVs	colorimetric	spectrum Analysis	artificial/human sweat	[117]
sweating rate	paper	semi-quantitative reading	calibration Method	artificial/human sweat	[133]
lactate, Na^+^, K^+^	CBVs	laboratory determination	laboratory determination	human sweat	[175]
vitamin C, Ca^2+^, Zn^2+^, Fe^3+^	CBVs	colorimetric	RGB	human sweat	[193]
Glucose, Lactate, Uric acid, Mg^2+^, pH	Paper	colorimetric	RGB	artificial/human sweat	[194]
Sweating rate, Cl^−^	Polymer microchannel	semi-quantitative reading, colorimetric	RGB, Calibration Method	artificial/human sweat	[201]

Human sweat is composed of various substances such as metabolites, electrolytes, and trace elements. By measuring the biomarkers in sweat, the physiological state of the body can be detected. Metabolites are mainly composed of lactic acid and glucose. Detecting sweat lactate and glucose can determine the body’s blood sugar levels and exercise endurance levels. Potassium ion, sodium ion, chloride ion, and pH are the main electrolytes in the body. The levels of potassium and sodium ions reflect the hydration status of the body, and detecting chloride ions can help diagnose cystic fibrosis. Abnormal pH values may be related to type II kidney stones. In addition, trace elements and sweat rate can help detect multiple physiological diseases in the body.

## 6. Challenges

While remarkable progress has been made in wearable sweat sensors, there remain several significant challenges across various aspects, such as continuous sweat sampling without contamination, reliable data reading, sensor lifespan, and user comfort.

Sampling is an essential component for sweat detection, responsible for gathering and transporting sweat samples to the detection area, where the sensing element is located. The sampling process, however, presents several formidable challenges, including the need to obtain a minimum amount of sweat, prevent the intermingling of fresh and aged sweat, and address concerns regarding sweat evaporation and leakage. Sponges and gauze are commonly employed to collect human sweat in previous research, which is time consuming and laborious [202,203]. In addition, when the sweat gathering method is not designed exquisitely, the collected sweat samples may be exposed to the atmosphere directly, making them prone to evaporation, contamination, leakage and re-adsorption [204,205]. In recent years, microfluidic technology, renowned for its capability to collect, transport and manipulate minute liquid, has been seamlessly integrated with sweat sensing methodologies by pioneering researchers [206,207]. This integration has led to the evolution of multifunctional sweat sensors, proficient in the continuous collection, renewal, and detection of minute sweat volumes. Consequently, these advancements have significantly elevated the capabilities of wearable sweat sensors.

The phenomenon of sweat sample evaporation should not be underestimated, as it can lead to an increase in analyte concentration, consequently yielding detection results that surpass the true values. This issue is particularly pronounced when dealing with minuscule sample volumes. In some instances, inadequate microfluidic chip design or unsuitable material selection can result in the unintended leakage and absorption of sweat samples. While this may have relatively minor repercussions on detection outcomes when ample sample volumes are available, it can substantially compromise sensitivity when working with limited sample quantities. Therefore, it is imperative to diligently address these aforementioned concerns.

Non-electrochemical sweat sensors, such as colorimetric, fluorescence and spectroscopy methods, determine the concentrations of target sweat biomarkers by measuring the optical properties of the reaction products formed between the reagents and sweat [208,209]. However, when dealing with a small sweat volume or insufficient reaction time, achieving complete reaction between the reagents and the sweat sample can be challenging. This challenge may lead to issues such as uneven color development within the detection area, potentially introducing bias in color information extraction and resulting in measurement inaccuracies [210,211]. The timely renewal of sweat samples is of paramount importance. Failure to do so can result in the mixing of fresh and aged sweat within the detection area, leading to measurement outcomes that represent an averaged value over a specific timeframe. Consequently, this may yield less timely and less precise detection data [81,83]. Furthermore, if the aged sweat becomes contaminated and is not promptly removed, it can even lead to significant deviations between the detected results and the actual values.

A common limitation of non-electrochemical measurement of sweat biomarkers lies in the inability to achieve continuous real-time measurements [80,104,105,107,112]. In non-electrochemical sweat sensors, data reading is discrete, and the timeliness of data acquisition relies on the frequency of sample testing. For example, when quantifying sweat biomarkers using colorimetric sweat sensors, the concentrations of these biomarkers in the sample are determined by comparing the color intensity of the reaction products to standard color scales [104,105]. Consequently, this implies that the data acquisition of colorimetric sweat sensors is discrete, and the timeliness of data is closely linked to the sampling frequency. Additionally, this method may introduce subjective errors into colorimetric analysis, as it relies solely on visual assessment of the color changes in the reagents. Individuals with color blindness or color vision deficiencies may encounter difficulties in visually interpreting the test results. Both fluorescence and spectroscopy techniques rely on the collection and identification of optical spectrum signals. Consequently, due to the constraint of low data acquisition frequency, it becomes challenging to acquire data with a high level of continuity. Furthermore, the acquisition of spectral information necessitates the use of additional excitation light to illuminate the reaction production of sweat sample and reagents, thereby diminishing the portability of wearable sensors [122,132,212].

There is ample room for further improvement in the accuracy of non-electrochemical methods for sweat analysis in several aspects. For instance, in colorimetric methods for the detection of sweat biomarkers, filter paper is commonly used as the substrate of colorimetric agents. However, due to the capillary and coffee-ring effects inherent to filter paper, the color unevenness within the colorimetric area can lead to variation in the extraction of color RGB information, subsequently resulting in detection errors [213]. In addition, the leaching and diffusion of the colorimetric reagent located in the detection zone can also result in spatially uneven color responses, decreasing the accuracy of the readings [107]. When extracting color information from images it is highly susceptible to environmental lighting conditions [76,90]. Different light intensities and varying color temperatures can introduce deviations in the extracted color features in images. Additionally, ambient light interference can impact the accuracy of the readings [76]. Enzymes are frequently employed in the analysis of sweat metabolites, and enzyme activity is susceptible to environmental conditions like temperature and pH [214]. Addressing the correction and calibration of these environmental factors remains a pressing concern.

The lifespan and comfort of wearable sweat sensors are also highly significant characteristics. Enzymes as a kind of catalyzer, can catalyze the oxidation of organic compounds and are widely used in non-electrochemical sensors such based fluorescence and colorimetry non-electrochemical sweat sensors. These enzyme-based sweat sensors can be easily influenced by the environment. For instance, enzyme activity can be impacted by environmental variables, such as temperature and pH, and enzyme inactivation can be induced when exposed in extreme conditions, like high temperatures, strong acids, and strong bases [214], Therefore, the monitoring performance of enzyme-based sensors severely declines in extreme environments, and even causes sensor scrap. Additionally, enzymes gradually lose their activity over extended periods of use. This reduction in enzyme activity can lead to a decrease in sensor sensitivity, impacting the accuracy and stability of the sensor, ultimately diminishing the sensor’s operational lifespan. For non-enzyme sensors, environmental factors, such as light, temperature, and humidity, can also impact the sensor’s lifespan. For instance, in the detection of sweat using colorimetric sensors, phenolphthalein and methyl orange are commonly used reagents for measuring sweat acidity and alkalinity. However, prolonged exposure to ultraviolet (UV) light can cause these reagents to fade in color. In the case of sweat sensors based on fluorescence methods, UV light can also lead to photobleaching of fluorescent molecules, resulting in a gradual weakening of the sensor’s signal response and thus reducing the lifespan of fluorescence sensors. Polydimethylsiloxane (PDMS) is a commonly used material for fabricating microfluidic devices, and researchers often employ a layer-by-layer stacking approach to assemble microfluidic devices [215]. However, this manufacturing process for wearable sensors significantly increases device thickness, causing discomfort when worn on the body. Additionally, polymer substrates typically exhibit poor breathability, which can considerably impede the natural respiration of the human skin. In addition, in the measurement process of non-electrochemical sweat sensors based on fluorescence and SERS, an external light source excitation device is often necessary. This device may need to be temporarily separated from the body’s skin, which can lead to discomfort. While fluorescence-based methods can use the light source and detection directly from a smartphone, they are susceptible to interference from ambient light. Therefore, it is common to use a shielding enclosure to block out external light during the measurement. This requires placing the smartphone and the shielding enclosure on the arm for testing, which can cause pressure on the skin and result in discomfort for the individual.

## 7. Conclusions and Future Work

Sweat, as a physiological fluid in the human body, has begun to show promising results in the fields of health assessment and disease diagnosis. Non-electrochemical methods, including colorimetry, fluorescence, and SERS, offer advantages such as small size, light weight, and low cost for the detection of sweat biomarkers. They are developing in parallel with electrochemical methods and are achieving significant milestones in this regard. This article provides an in-depth exploration of the recent advancements in non-electrochemical sweat sensors, covering various aspects including sampling methods, detection techniques, and signal readout. It further discusses the practical applications of these sensors in measuring crucial biomarkers present in sweat. The article also summarizes the current bottlenecks and challenges of non-electrochemical sweat sensors.

The accurate measurement of sweat requires sufficient and fresh sweat samples. The sampling methods mainly include paper-based non-electrochemical sweat sensors, thread-based non-electrochemical sweat sensors, and non-electrochemical sweat sensors based on microfluidic valves. Paper-based sensors have numerous capillaries, which can quickly collect and transfer sweat [68,69]. Thread exhibits superior toughness compared to paper-based materials, enabling it to endure higher levels of stress without breaking [79,216,217]. Microfluidic technology mainly uses microvalves to manipulate the flow of liquids and achieve precise timed sampling [92,93].

After the liquid reaches the detection area, the concentration of the target substance is analyzed using non-electrochemical methods such as colorimetry, fluorescence, and spectroscopy [104,121,133]. Colorimetry analyzes the concentration of the target substance by observing the color change of the colorimetric reagent without the need for an external light source, but it has a limited detection range [106,218]. Fluorescence, requiring external light, measures intensity changes for concentration calculation, enhancing sensitivity and detection speed [52,53,218,219]. SERS quantitatively analyzes the concentration of the target substance by measuring the Raman scattering of the target substance. SERS requires the participation of an external light source and has high sensitivity and stability [130,132]. In addition, reference marks such as scales can be set up on the sensor, and data can be directly read using the naked eye. 

The method of reading signals significantly impacts data accuracy, with options like RGB, chromaticity diagram, and spectrum analysis. For colorimetry, the target substance concentration is analyzed by measuring a single value of R, G, or B in RGB [101,102,103]. However, for some substances, a single value may not suffice, necessitating consideration of chromaticity coordinates [137,138]. Fluorescence and SERS methods quantitatively analyze the concentration of the target substance by measuring the wavelength or peak of the target substance in the detection area. Additionally, in the absence of electronic devices, semi-quantitative readings can be obtained using the naked eye. Biomarkers in sweat are correlated with those in blood. Analyzing the biomarkers and physical parameters (such as sweat rate) in sweat, such as metabolites (lactate, glucose, cortisol, etc.), electrolytes (sodium, potassium, chloride, etc.), and trace elements (zinc, magnesium, iron, etc.), can help to analyze the physiological condition of the human body.

Although non-electrochemical sensors have made significant progress, there are still many shortcomings to be solved. Sampling faces many challenges, and one of the biggest challenges for sweat analysis is the need for a sufficient volume of sweat for analysis. The amount of sweat secreted by the body at rest is minimal and cannot meet the fluid volume required for sensor detection. Exercise can cause the body to secrete a large amount of sweat, but exercise requires a large amount of energy from the body, in addition some people who have difficulty moving are unable to engage in intense physical activity. The use of sweating techniques, such as iontophoresis [214,220] can stimulate sweat production in the body, but this method requires external electrical power, which may cause skin irritation due to the small electric current flowing on the skin surface. Osmotic pressure [221] is a natural phenomenon caused by the imbalance of solute-solvent chemical potential between two solutions separated by a semipermeable membrane. The high concentration diffuses to the low concentration until the difference is eliminated. Based on the principle of osmotic pressure, the use of hydrogels [222] can extract sweat from the epidermis to the outside, achieving non-invasive sweating and providing sufficient samples for sweat sensors. The mixture of old and new sweat can easily cause errors in the detection results. The use of microfluidic valves [90,92] can precisely manipulate the liquid and achieve timed sampling to avoid mixing old and new sweat. In addition, setting up an evaporation zone in the microfluidic device can use liquid evaporation to accelerate the flow of liquid in the microchannels and achieve timely updating of sweat.

The detection of non-electrochemical sensors is discrete, and it is unable to read data continuously and in real-time. Electrochemical sensors employ the principles of electrochemistry to detect the concentration of chemical substances [223,224,225]. They do so by measuring the current or potential changes generated in response to electrode reactions, which are then correlated with substance concentrations. This enables continuous measurement. Combining non-electrochemical sensors with electrochemical sensors can compensate for the inability of non-electrochemical sensors to continuously detect [226]. The detection accuracy of non-electrochemical sweat sensors is easily affected by external light sources. Some researchers use isolation hoods to shield the microfluidic device from external light interference during detection [117]. However, this method is cumbersome, so some researchers focus on signal processing and establish compensation equations to eliminate the effects of illumination conditions [62]. In addition, enzyme-based sensors are susceptible to temperature and pH interference. The use of compensation functions can calibrate measurement results [227,228,229].

The lifespan and comfort of sweat sensors are equally important [208,230,231]. Most microfluidic devices are disposable and have a short lifespan, mainly because of the accumulation of sweat in the microfluidic device. In the future, detachable devices can be repeatedly used, which are expected to enhance the device’s lifespan. Additionally, microfluidic devices with sample discharge function will further increase the lifespan of sweat sensors [231]. Conventional microfluidic devices are typically fabricated by employing polymers to construct separate functional layers, which can cause discomfort to the human body [232,233,234]. However, paper-based and thread-based materials offer a remarkable combination of liquid transport capability, lightweight nature, breathability, and cost-effectiveness [235,236]. Consequently, these materials are poised to emerge as the predominant choice for sweat sensors. The non-electrochemical sweat sensor enables non-invasive health monitoring, providing a solution for early disease diagnosis and management. This method overcomes the limitations of traditional laboratory tests and allows for real-time detection. However, there are still many challenges and issues to be addressed, such as sampling, interference resistance, lifespan, and comfort. Further research in this area can accelerate the development of wearable sensors, and non-electrochemical sweat sensors have broad application prospects in health monitoring, disease diagnosis, and drug development, providing convenient, accurate, and reliable solutions.

Significant progress has been made in the field of wearable devices, including commercialized smartwatches, smart wristbands, portable hydration monitors, and fitness trackers. As for sweat sensors, they are currently in the nascent stages of commercialization, with numerous technical and business challenges yet to be addressed. For instance, establishing accurate correlations between sweat biomarkers and human health requires extensive clinical data support. Additionally, addressing individual variations in sweat detection necessitates the integration of artificial intelligence and big data analysis to enhance the sensor’s personalization capabilities. Furthermore, sweat sensors contain a wealth of private physiological health information, such as individual health conditions, metabolic states, and activity patterns. Manufacturers and research institutions must implement effective privacy protection measures to ensure the proper safeguarding of user privacy. Simultaneously, relevant regulations and ethical guidelines need gradual refinement to secure users’ privacy rights in the application of sweat sensors. Successfully advancing the commercialization of such sensors requires collaborative efforts from various stakeholders, including researchers, healthcare professionals, and suppliers.

## Figures and Tables

**Table 1:** Comparation of the advantages and disadvantages in detecting sweat biomarkers using electrochemical and non-electrochemical methods.

Aspect	Electrochemical	Non-Electrochemical Sensors
Sensitivity	High sensitivity	Medium to high sensitivity, the sensitivity range is often determined by the testing method.
Selectivity	Strong selectivity	Selectivity depends on the sensor type and technology, often with strong selectivity.
Response time	Generally quick response time	Response time varies and can be slower in some cases.
Detection range	Wide detection range for certain analytical substances	Wide detection range for various analytical substances.
Accuracy	Moderate accuracy	Accuracy depends on sensor type and calibration.
Interference	Susceptible to interference from other substances.	Less susceptible to interference from non-target objects.
Power Consumption	High power consumption.	Lower or even zero power consumption.
Size and Portability	Large size, even when worn on the human body, there is still a strong sense of foreign objects.	Small size, easy to carry, and more comfortable.
Weight	Medium weight	Light weight
Cost	Requires a large number of electronic devices, high cost.	No additional electronic devices are required, low cost.
Maintenance	Regular calibration and maintenance are required.	Lower maintenance, but periodic calibration.
Lifespan	The service life of components such as electrodes and batteries are limited.	Most have a longer lifespan, but in some low-cost solutions, the device is disposable.
